# Supplementary material for: Asymmetric interactions between doublesex and tissue- and sex-specific target genes mediate sexual dimorphism in beetles
Source: Nat Commun. 2017 Feb 27;8:14593. doi: 10.1038/ncomms14593 (PMC5333360; doi:10.1038/ncomms14593)
Supplement: Supplementary Information — Supplementary Figures [file ncomms14593-s1.pdf]

Supplementary Figure 1: Distribution of genes with sex-biased expression across tissues.

Among tissues within sexes, the majority of genes with sex-biased expression are uniquely expressed in genital tissue. The remaining genes with sex-biased expression are concentrated in single tissues in both sexes (e.g., 26% of genes with male-biased expression are expressed exclusively in head horn tissue).

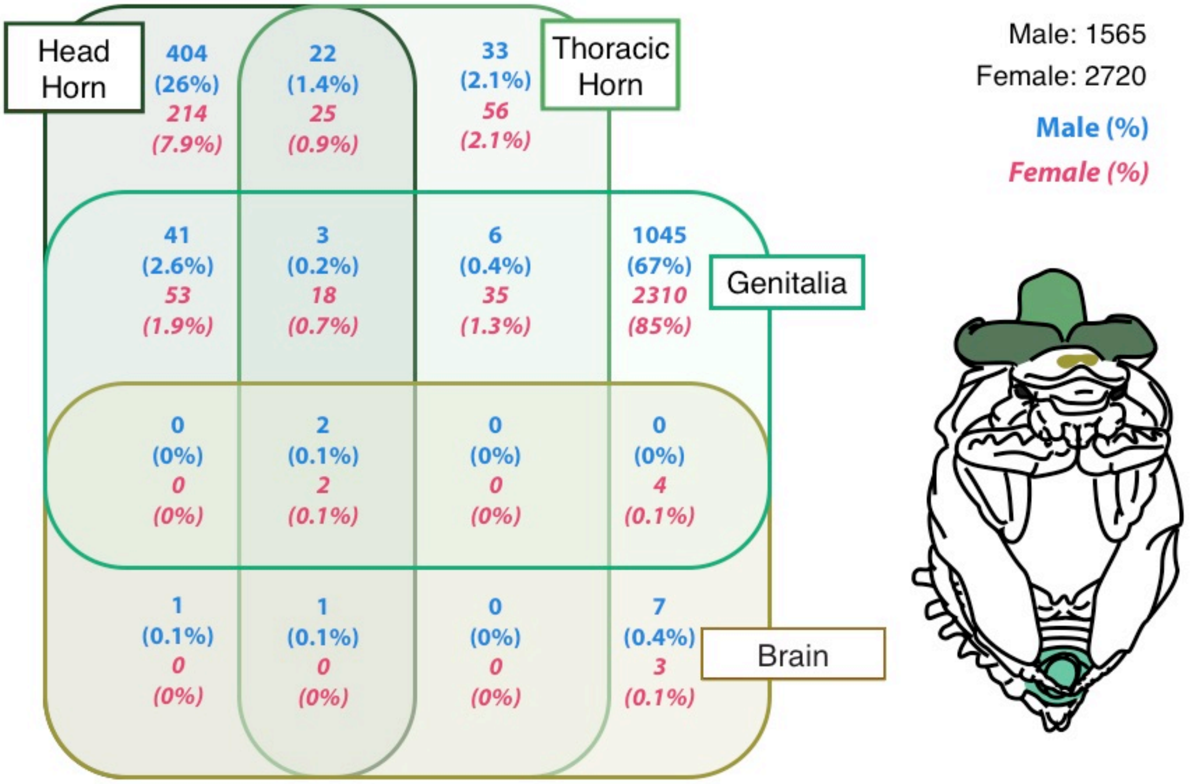

Supplementary Figure 2: *dsx*RNAi modulates male- and female-biased gene expression in a sex-specific and tissue-specific manner. Dark blue and pink represent genes with male- and female-biased expression, respectively, as determined by comparisons between control males and control females. Background colors indicate whether those genes were assessed in male (light blue) or female (light pink) tissues. Letters indicate significant differences between levels of gene expression (Wilcoxon test and Benjamini-Hochberg correction for multiple comparisons). **(A)** In male brain and genitalia, *dsx*RNAi decreased levels of male-biased gene expression and increased levels of female-biased gene expression. In female brain and genitalia, *dsx*RNAi neither decreased levels of female-biased gene expression nor increased levels of male-biased gene expression. **(B)** A summary of the differential actions of male and female Dsx isoforms among tissues; dark blue arrows indicate the effect on genes with male biased expression, dark pink arrows indicate the effect on genes with female-biased expression, and gray arrows indicate the absence of an effect.

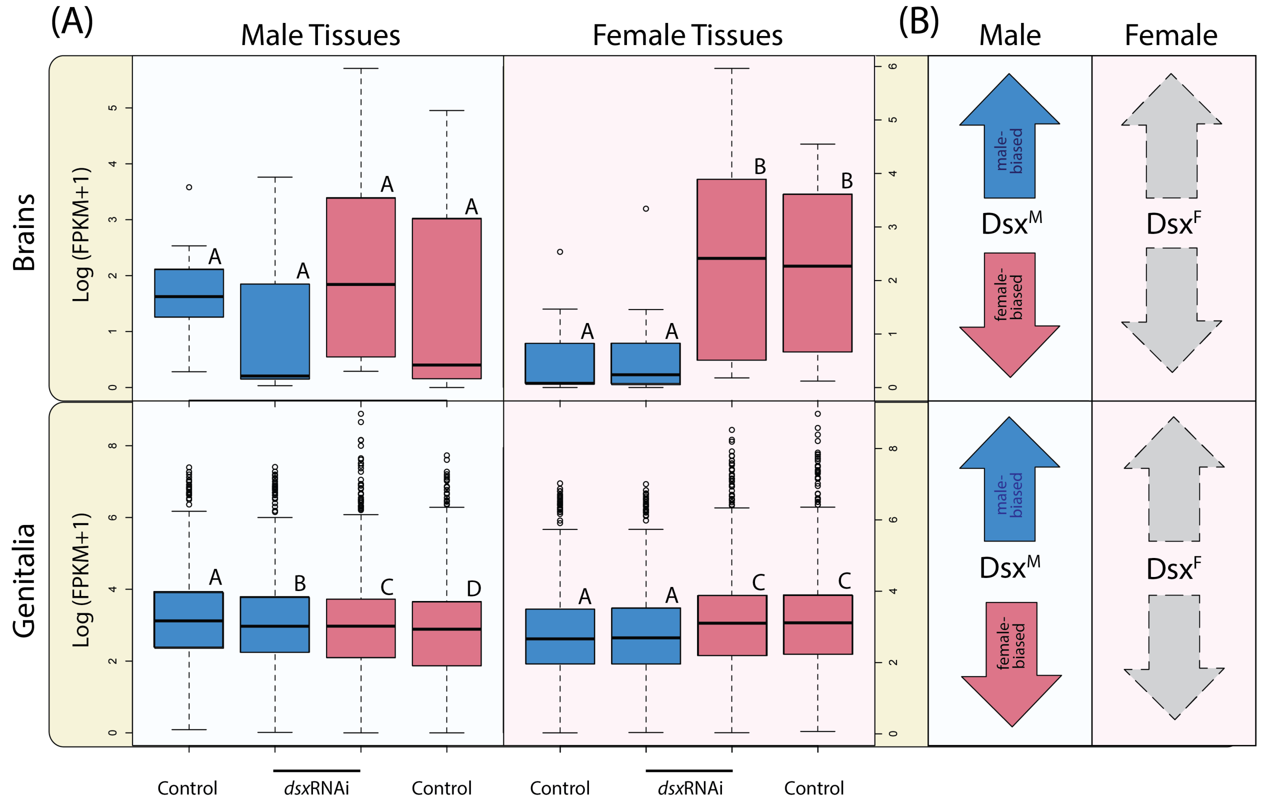

Supplementary Figure 3. Distribution of Dsx targets across tissues.

Among tissues within sexes, the majority of Dsx targets are uniquely expressed in head horn tissue. The remaining Dsx targets are concentrated in single tissues in both sexes (e.g., 16% of male Dsx targets are expressed exclusively in thoracic horn tissue), with the only major divergence between males and females being that male head and thoracic horn tissues share a sizeable number of Dsx targets (11% of all male targets).

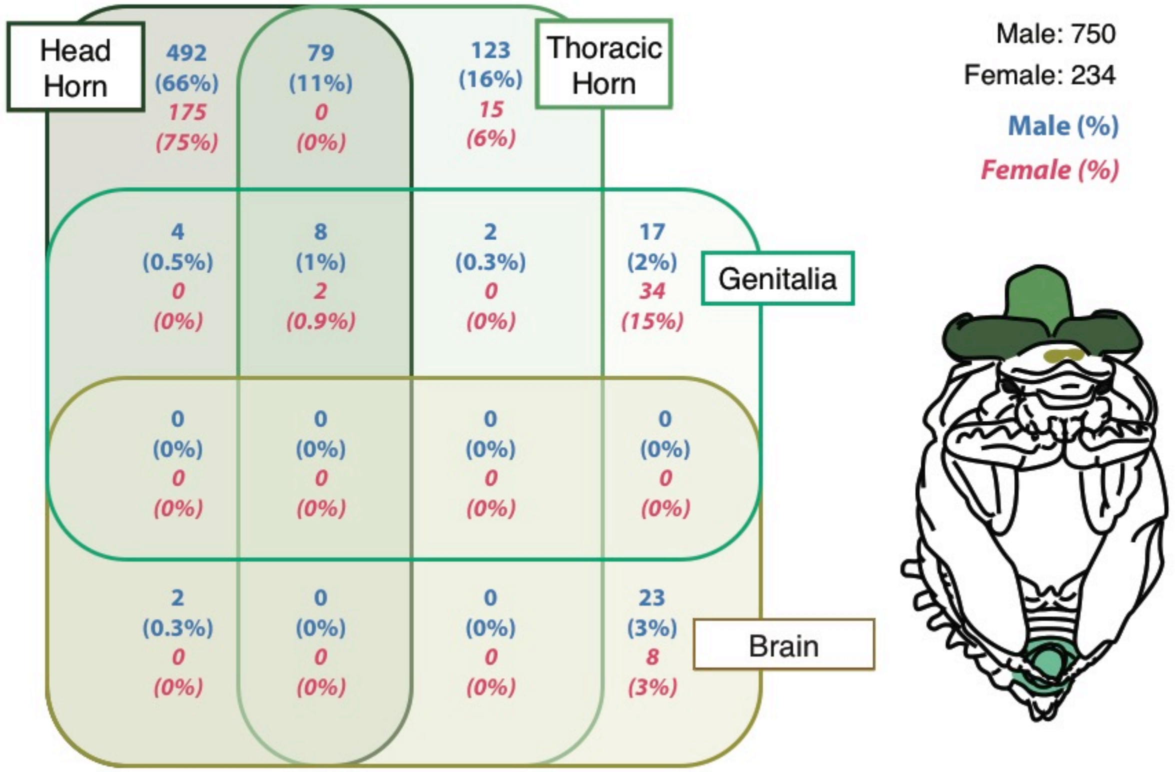

Supplementary Figure 4: *dsx*RNAi Efficiency

FPKM (fragments per kilobase of transcript per million mapped reads) values for *dsx*, estimated from RNA-Seq data, were used to determine the efficiency of our *dsx*RNAi treatment. We modeled the log-transformed fold change in *dsx* FPKM values as a function of both sex and treatment (control or *OtdsxC* dsRNA injection). For tissues in which data met the requirement of normality and homoscedasticity (head horns and genitalia), an ANOVA was used; when such requirements were not met (thoracic horns and brains), Wilcoxon tests were used, followed by Benjamini-Hochberg corrections for multiple comparisons.

We found that while *OtdsxC* dsRNA injections were successful at significantly knocking down *dsx* gene expression in male and female headhorns, thoracic horns and genitalia, but not brain. Failure to observe a significant effect of *dsx*RNAi in brain could be due to *dsx* baseline levels being already much lower than in the other tissues assessed, or due to the existence of compensatory mechanisms specific to this tissue (*** indicates a p-values <0.005, **indicates p-values <0.05, and * indicates a p-value = 0.06).

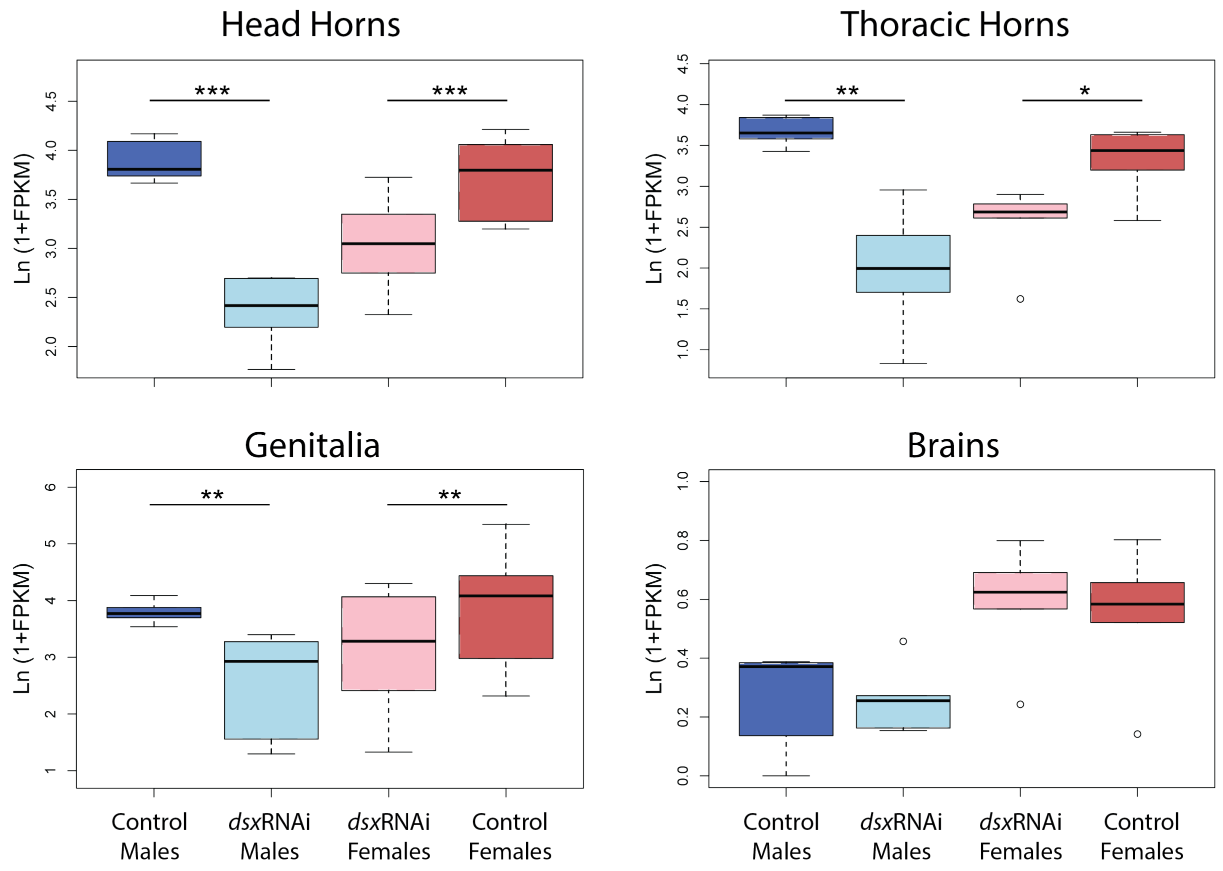

Supplementary Figure 5: Correlations between biological replicates

A.

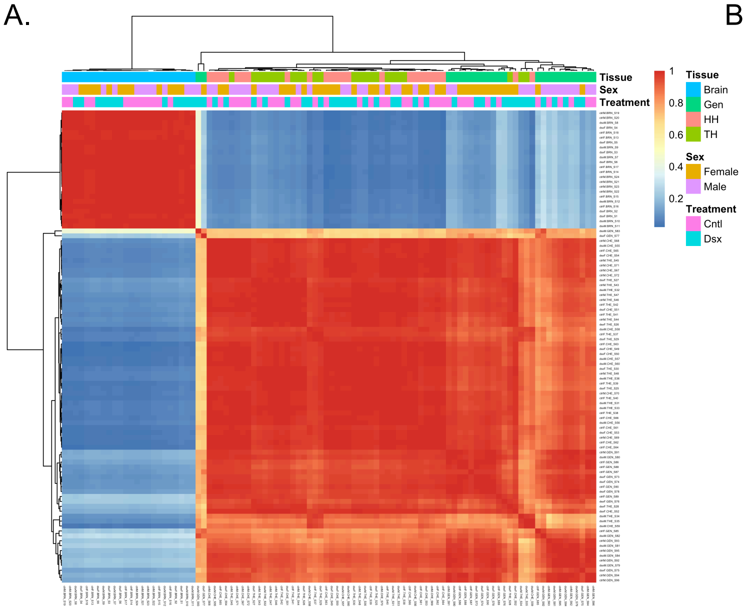

B.

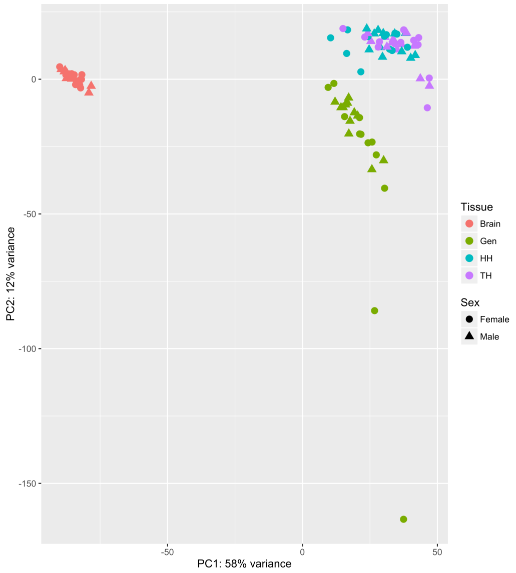

C.

Biological Replicate Pearson's Correlations

Tissue	Sex	Treatment	Min_Correlation	Max_Correlation	Mean_Correlation	Min_P_value	Max_P_value
Brain	Male	Control	0.9927	0.9966	0.9948	0	0
Genitalia	Male	Control	0.8966	0.9935	0.9605	0	0
Head Horns	Male	Control	0.8102	0.9955	0.9337	0	0
Thoracic Horns	Male	Control	0.8027	0.9945	0.9271	0	0
Brain	Male	dsxRNAi	0.9804	0.9949	0.9886	0	0
Genitalia	Male	dsxRNAi	0.4236	0.9869	0.7402	0	0
Head Horns	Male	dsxRNAi	0.4243	0.9961	0.74	0	0
Thoracic Horns	Male	dsxRNAi	0.3716	0.9886	0.7095	0	0
Brain	Female	Control	0.9825	0.9961	0.9904	0	0
Genitalia	Female	Control	0.5376	0.9753	0.8364	0	0
Head Horns	Female	Control	0.5467	0.9916	0.8695	0	0
Thoracic Horns	Female	Control	0.4524	0.9878	0.8388	0	0
Brain	Female	dsxRNAi	0.9901	0.9958	0.9892	0	0
Genitalia	Female	dsxRNAi	0.3806	0.9744	0.7749	0	0
Head Horns	Female	dsxRNAi	0.3569	0.9688	0.759	0	0
Thoracic Horns	Female	dsxRNAi	0.3212	0.9727	0.7467	0	0

FPKM (Fragments Per Kilobase of transcript per Million mapped reads; i.e., gene expression) values were used to generate a (A) heatmap and (B) PCA plot in order to visualize correlations among biological replicates. Additionally, a correlation matrix of Pearson's correlation coefficients of gene expression between samples was generated (Supplementary data 6), along with p-values for those correlations. P-values were calculated using a two tailed t-test, and were adjusted for multiple tests using Holm's correction. The minimum, maximum and mean pairwise correlation coefficients between biological replicates are summarized above (C). Given the nature of the tissues and RNAi treatments assessed, some variation in gene expression between biological replicates was expected. Nevertheless, in all cases, the mean correlation between biological replicates exceeded 0.70, the maximum correlation between biological replicates exceeded 0.95, and the significance of correlations between biological replicates was highly significant ($p<0.01$).
